# Different Amounts of Nitrogen Fertilizer Applications Alter the Bacterial Diversity and Community Structure in the Rhizosphere Soil of Sugarcane

**DOI:** 10.3389/fmicb.2021.721441

**Published:** 2021-09-10

**Authors:** Yan Gu, Jihua Wang, Weijun Cai, Guoliang Li, Yu Mei, Shaohai Yang

**Affiliations:** ^1^Guangdong Provincial Key Laboratory of Crops Genetics and Improvement, Crop Research Institute, Guangdong Academy of Agricultural Sciences, Guangzhou, China; ^2^Zhanjiang Academy of Agricultural Sciences, Zhanjiang, China; ^3^Institute of Agricultural Resources and Environment, Guangdong Academy of Agricultural Sciences, Guangzhou, China

**Keywords:** 16S rRNA, bacterial community, rhizosphere microbe, nitrogen fertilizer, sugarcane

## Abstract

Sugarcane cropping systems receive elevated application of nitrogen (N) fertilizer for higher production, which may affect production costs and cause environmental pollution. Therefore, it is critical to elucidate the response of soil microbial to N fertilizer inputs in sugarcane soil. A field experiment was carried out to investigate the effects of optimum (N375, 375 kg N/ha) and excessive (N563, 563 kg N/ha) amounts of N fertilizer on soil bacterial diversity and community structure in a sugarcane cropping system by MiSeq high-throughput sequencing; 50,007 operational taxonomic units (OTUs) were obtained by sequencing the 16S rRNA gene amplicons. Results showed that the most abundant phyla in the sugarcane rhizosphere soil were *Proteobacteria*, *Actinobacteria*, *Acidobacteria*, and *Planctomycetes*, whose ensemble mean accounted for 74.29%. Different amounts of N application indeed change the bacterial diversity and community structures. Excessive application of N fertilizers significantly decreased the pH and increased the available N in soils and unexpectedly obtained a lower yield. Excessive N resulted in a relatively lower bacterial species richness and significantly increased the relative abundance of phyla *Proteobacteria*, *Acidobacteria*, and *Bacteroidetes* and the genera *Sphingomonas* and *Gemmatimonas*, while optimum N treatment significantly increased the phylum *Actinobacteria* and the genera *Bacillus* and *Nitrospira* (*P* < 0.05). N application shifted the N cycle in nitrification, mainly on the *Nitrospira*, but showed no significant effect on the genera related to nitrogen fixation, methane oxidation, sulfate reduction, and sulfur oxidation (*P* > 0.05). Overall, the optimum amount of N application might be conducive to beneficial microorganisms, such as *Actinobacteria*, *Nitrospira*, and *Bacillus* and, thus, result in a healthier ecosystem and higher sustainable crop production.

## Introduction

Sugarcane (*Saccharum* L. spp.) is an important agroeconomic sugar crop which is planted in over 20 million hectares worldwide (Val-Moraes et al., 2016). Sugarcane contributed into more than 90% of the total sugar production and has been considered one of the most promising crops for generating renewable bioenergy, which is expected to become the second largest energy source in the world by 2030 (Li Y. R. et al., 2016). China is one of the major sugar-producing countries in the world, producing more than 10 million tons of sugar annually (Li and Yang, 2015). Due to continuously increasing consumption, there is a need to increase sugarcane production to meet the growing demand.

Sugarcane is a fast-growing large biomass crop which requires large amounts of nutrients and water. Nitrogen (N) is the essential macronutrient required for sugarcane growth and development (Yang et al., 2013). In order to obtain higher crop yield, large amounts of N fertilizers were applied to provide nutrients for growing sugarcane, and N fertilizer application rate has increased several times in the past decades worldwide (Robertson and Vitousek, 2009; Zhang et al., 2013; Li and Yang, 2015). N fertilizer application for sugarcane production varies largely between countries, ranging from 60 kg/ha in some regions of Brazil to 755 kg/ha in some parts of China (Robinson et al., 2011). In China, N fertilization varies from 300 to 800 kg/ha N in sugarcane production, which is 3–10 times higher than in other countries (Li and Yang, 2015). However, excessive application of N fertilizer not only causes waste of resources and higher production cost but also results in serious environmental pollution and ecological environment degeneration (Waclawovsky et al., 2010; Belén et al., 2016). Furthermore, superfluous N fertilizer has adverse effect on sugar quality and often leads to a substantial decrease of beneficial microflora related to N mineralization (Singh et al., 2020).

Microbes in the rhizosphere play important roles in nutrient cycling and acquisition. Soil microorganisms form complicated microbial communities that regulate the nutrient cycles and influence soil characteristics, plant growth, and ecosystem sustainability (Singh et al., 2017). In turn, agronomic practices, such as fertilization can alter soil physical and chemical properties and, consequently, soil microbiomes. The effect of N fertilizer application on soil microbial community composition may be caused by direct effects of N nutrient or by indirect changes in soil and plant properties (Klironomos et al., 2011). Many studies have been undertaken to explore the effects of N fertilizer on microbes in agriculture ecosystems. Du et al. (2019) found that application of inorganic N fertilizer resulted in distinctive changes on N-cycle microorganism. Moreover, applying N fertilizer influenced soil microbial composition, particularly fungal community (Guo et al., 2018). A recent meta-analysis demonstrated that N fertilizers decreased both soil microbial diversity and the relative abundances of *Actinobacteria* and *Nitrospirae* but did not significantly change the primary fungal groups (Wang et al., 2018).

During the sugarcane life cycle, the plants and its roots establish associations with various diversities of microorganisms, including beneficial, detrimental, or neutral microbes. Several studies expectedly have been conducted to reveal the microbial diversity and community in sugarcane rhizosphere soil. Savario and Hoy (2011) have demonstrated that sugarcane monoculture can affect the composition of the microbial community in field soil by evaluating the culturable microorganisms using community level physiological profiles (CLPP). A culture-independent approach using the 16S rRNA gene library sequencing also has been used to assess the bacterial community in the rhizosphere soil of sugarcane under different nitrogen fertilization conditions. Pisa et al. (2011) found that *Proteobacteria*, *Acidobacteria*, *Bacteroidetes*, *Firmicutes*, and *Actinobacteria* were the predominant phyla in the rhizosphere soil of sugarcane. However, previous studies did not reveal the microbial community structure very well due to poor sequencing technologies. Recent studies on microbials in sugarcane soils mainly focused on plant growth-promoting bacteria, endophytic bacteria, and fungi or functional bacteria associated with sugarcane, such as nitrogen-fixing bacteria, aiming at exploring the massive potential of biofertilizer to replace the chemical N fertilizer (de Souza et al., 2016; dos Santos et al., 2019; Pereira et al., 2019; Singh et al., 2020). Yeoh et al. (2016) found that regulating N fertilizer rates does not improve sugarcane yields by enriching diazotrophic populations and optimal N fertilizer crops had higher biomass and higher abundances of nitrification and denitrification genes. The above studies highlighted that a deep understanding of how N fertilizer application affects microbial communities is important for achieving a balance in maximizing crop yields and minimizing nutrient pollution associated with N fertilizer application.

Despite the wide plantation and economic importance of sugarcane, knowledge regarding the microbial diversity and community of sugarcane rhizosphere soils is limited. Meanwhile, there still lacks a deep understanding of how N fertilization regimes affect the sugarcane yields and rhizosphere soil microbiome. Therefore, in our work, a field experiment was conducted to evaluate the impacts of different amounts of N fertilizers on the diversity and community structure in the sugarcane rhizosphere soil. The main objectives were to (i) reveal the bacterial diversity and community structure in the rhizosphere soil of sugarcane and (ii) evaluate the effect of N application amount on the soil physicochemical characteristics and crop yields and on the rhizosphere bacterial diversity and community structure. Moreover, we hypothesized that (iii) N fertilizer impact on sugarcane yield may be through the regulation of rhizosphere bacterial community and functions. The results of our work may provide guidance for the reasonable fertilization management of sugarcane fields, so as to promote sustainable development of the sugarcane industry.

## Materials and Methods

### Site Description and Experiment Design

The field trial was located in Mazhang district (21°15′36″N, 110°16′48″E) of Zhanjiang, Guangdong Province, China. The area has a south subtropical monsoon climate, with an annual mean precipitation and temperature of 1,800 mm and 23.5°C, respectively. Land use was peanut and sweet potato rotation before and has been transformed to cultivate sugarcane since 2016. The experiments were conducted from 2016 with three different N fertilization rates in new planted sugarcane for 2 years. The experimental field was divided into plots of 30 m^2^ (5 m × 6 m), and each treatment was performed in triplicate with a randomized block design from February to December. Four treatments were included in this study. The CK treatment was without fertilizers and the other three annual N fertilization regimens were applied with urea (46% N) as follows: N0 (0 kg N/ha), N375 (375 kg N/ha), and N563 (563 kg N/ha); 375 kg N/ha was the appropriate N fertilizer amount and 563 kg N/ha was the average N application of farmers after preliminary investigation and research. Phosphorus (P) and potassium (K) were supplied with equal amounts of 112.5 and 375 kg/hm^2^ in the form of calcium superphosphate (12% P_2_O_5_) and potassium chloride (60% K_2_O). N, P, and K were applied in three split doses in basal, tillering stage, and elongation stage at ratios of 5:8:7, 1:1:1, and 1:15:6, respectively. Field management was carried out by local cultivars and conventional crop practices.

### Soil Sampling and Analysis of Physicochemical Properties

On February 20, 2018, rhizosphere soil samples were obtained by manually shaking the loosely attached soil from the roots, 1 week after sugarcane was harvested. The replicated samples were pooled in polyethylene self-sealing bags and then immediately transported to the laboratory in a container with enough dry ice. Each sample was a composite formed by mixing together the eight subsamples and then divided into two aliquots. One aliquot was stored at −4°C for subsequent biochemical analyses as soon as possible within 1 week. Another aliquot was stored at −80°C for DNA extraction and sequencing.

Several soil physicochemical properties were determined. Soil pH was determined with a suspension of soil/water (w/v) ratio at 1:2.5 by a glass electrode pH meter. Soil organic carbon (SOC) was determined using K_2_Cr_2_O_7_ wet oxidation and titration by FeSO_4_, and organic matter (OM) was converted from SOC (Bao, 2000). Alkali-hydrolyzable N (AN) was determined by alkali hydrolysis diffusion method. Available phosphorus (AP) measurement was extracted with HCl-NH_4_F and determined using the phosphomolybdate blue colorimetric method. Available potassium (AK) was measured by flame photometry after ammonium acetate extraction.

### Soil DNA Extraction, PCR Amplification, and Illumina Sequencing

Soil DNA was extracted from 0.50 g sample soil with E.Z.N.A. stool DNA Kit (Omega Bio-Tek, Norcross, GA, United States) according to the protocols of the manufacturer. A NanoDrop 2000c UV–Vis spectrophotometer (Thermo Fisher Scientific, Waltham, MA, United States) was used to check the DNA quality and quantity. DNA extracts were stored at −20°C until analysis.

The purified DNA was used as a template for amplifying the V3–V4 region of the 16S rRNA gene with the barcode primer set 341F (5′-CCTACGGGNGGCWGCAG-3′)/806R (5′-GGACTACHVGGGTATCTAAT-3′). The thermal cycle conditions were as follows: initial denaturation at 95°C for 2 min, followed by 27 cycles of 98°C for 10 s, 62°C for 30 s, and 68°C for 30 s, and a final extension at 68°C for 10 min. PCR amplification was carried out in a total of 50 μl reaction system containing 100 ng of template DNA, 1.5 μl of 5 μM forward and reverse primers, 1 μl KOD DNA polymerase, 5 μl of 10 × KOD buffer, and 5 μl of 2.5 mM dNTP mixture. Amplicons were extracted from 2% agarose gels and purified using the AxyPrep DNA Gel Extraction Kit (Axygen Biosciences, Union City, CA, United States) and quantified using a QuantiFluor fluorimeter-ST (Promega, Madison, WI, United States). Purified amplicons were pooled in equimolar concentrations and paired-end sequenced (2 × 250) on an Illumina HiSeq platform by Illumina NovaSeq 6000 according to standard protocols (Chen et al., 2019). Sequencing of the 16S rRNA gene was performed in Gene *Denovo* Biotechnology Co., Ltd. (Guangzhou, China).

### Data Processing and Bioinformatics Approaches

Raw data containing adapters or low-quality reads [containing more than 10% of unknown nucleotides (N) or less than 80% of bases with quality (*Q*-value) > 20] were trimmed, after which pair-ended reads were merged into one sequence as raw tags using FLASH (version 1.2.11) with a minimum overlap of 10 bp and mismatch error rates of 2%. Raw tags were quality-filtered and processed using the Quantitative Insights into Microbial Ecology (QIIME) (version 1.9.1) according to the following three criterions (Caporaso et al., 2010). The clean tags were aligned in the Gold database and reference-based chimera checking was performed using the UCHIME algorithm to identity and eliminate putative chimeric sequences. The obtained effective tags were clustered into operational taxonomic units (OTUs) of ≥97% sequence similarity using UPARSE (Edgar, 2013). The representative sequences classified into organisms by a naive Bayesian model using the RDP classifier (version 2.2) based on the SILVA database in the main OTUs were analyzed using BLAST with the NCBI database to obtain the most similar published sequences (Gu et al., 2019). Finally, the complete dataset was deposited into the NCBI Sequence Read Archive (SRA) database under accession number SRP269446.

### Statistical Analysis

A Venn diagram was conducted to compare the OTUs among the soil samples. The richness and evenness analysis based on OTU was performed to assess the biodiversity of microbial communities in different N fertilizer-applied soils. Duncan’s multiple range test was employed to compare statistically significantly differences (*P* < 0.05) of the alpha-diversity indices and among different treatments. Principal coordinate analysis (PCoA) and the unweighted pair group method with arithmetic mean (UPGMA) clustering analysis based on Bray–Curtis were used to assess similarities and discrepancies of the bacterial community structure among all treatments. Differences in functional groups between fertilizer treatments were determined using one-way analysis of variance (ANOVA). The linear discriminant analysis effect size (LEfSe) method was used to identify the biomarkers of soil bacteria among the treatments (Segata et al., 2011). Permutational multivariate analysis of variance (PERMANOVA) was performed using R software (version 3.6.3) to test the differences in community composition based on the Bray–Curtis distance.

## Results

### Soil Physicochemical Characteristics and Crop Yield

The selected physiochemical characteristics of the soil samples are presented in Table 1. The yield of sugarcane was 107.75 t/ha in the N375 treatment, significantly higher than 102.11 t/ha in the N563 treatment and 96.24 t/ha in the N0 treatment (*P* < 0.05). The N563 treatment has the lowest pH and the highest organic matter and AN among all the treatments. There were no significant differences on available P and K between the N375 and N563 treatment (*P* > 0.05).

**TABLE 1:** Physiochemical properties and crop yields in all the treatments.

**Treatments**	**pH**	**Organic matter (g/kg)**	**Alkali-hydrolyzable N (mg/kg)**	**Available P (mg/kg)**	**Available K (mg/kg)**	**Yield (t/ha)**
						
CK	4.560.06a	13.770.25c	82.475.90b	47.4530.50b	80.5012.50b	84.133.15d
N0	4.380.24ab	14.290.44bc	80.534.80b	231.0914.30a	117.6711.10a	96.242.06c
N375	4.280.02b	14.700.47ab	89.021.10b	240.5626.00a	116.5020.10a	107.750.21a
N563	4.030.04c	15.270.61a	117.644.80a	251.9919.30a	127.839.90a	102.111.29b

*Values represent mean ± standard deviation of triplicate measurements.*

*Different lowercase letters in the same column indicate significant differences (*P* < 0.05) among the four treatments.*

*Yields were the average of 2 years.*

*CK, without fertilizers; N0, without N fertilization; N375, application of 375 kg/ha N; N563, application of 563 kg/ha N.*

### Soil Bacterial Community Diversity

**FIGURE 1:**
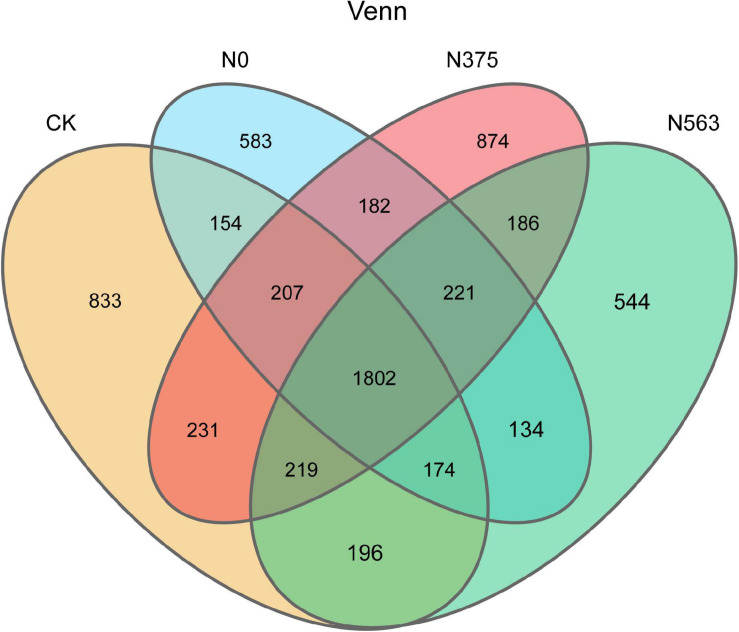
A Venn diagram demonstrating the unique and common bacterial OTUs among the four treatments. CK, without fertilization; N0, without N fertilization; N375, application of 375 kg/ha N; N563, application of 563 kg/ha N.

A total of 620,112 reads with an average valid sequence length of 420 bp and 50,007 OTUs were obtained from the four treatments (including 12 soil samples) (Table 2). The similarities and differences among OTUs of the four treatments are shown in a four-set Venn diagram (Figure 1). The unique OTUs were 833, 583, 874, and 544 for the CK, N0, N375, and N563 treatments, respectively, and the four treatments shared 1,802 OTUs.

**TABLE 2:** Species richness and diversity indices of the four treatments at a 97% identity threshold.

**Treatments**	**Total tags**	**OTU numbers**	**Good**’**s coverage**	**Observed species**	**Shannon index**	**Chao1**	**ACE**
CK	45,3754,389	4,190393	0.9630.003	4,088298ab	9.72610.2646a	5,583.98424.41ab	5,558.58451.11ab
N0	46,7723,071	3,937194	0.9640.002	3,803123ab	9.42480.1754a	5,394.90184.26ab	5,308.22276.03ab
N375	55,3043,117	4,646413	0.9600.003	4,223299a	9.69860.2549a	5,932.30420.95a	5,974.41420.58a
N563	48,8593,547	3,896313	0.9670.004	3,706259b	9.57140.0979a	5,100.39451.63b	5,070.81489.81b

*Values represent mean ± standard deviation of triplicate measurements.*

*Different lowercase letters in the same column indicate significant differences (*P* < 0.05) among the four treatments.*

*CK, without fertilization; N0, without N fertilization; N375, application of 375kg/ha N; N563, application of 563 kg/ha N.*

Good’s coverage indices for all samples were above 0.96, indicating that the sequencing depth was large enough to capture the complete diversity of each sample. The Shannon indexes used to describe the community diversity showed no significant differences among the four treatments (*P* > 0.05). However, the indices including observed species, Chao1, and ACE of the N563 treatment were significantly lower than the N375 treatment (*P* < 0.05), indicating that excessive application of N fertilizer resulted in a relatively lower bacterial species richness.

### Soil Bacterial Community Dissimilarity

**FIGURE 2:**
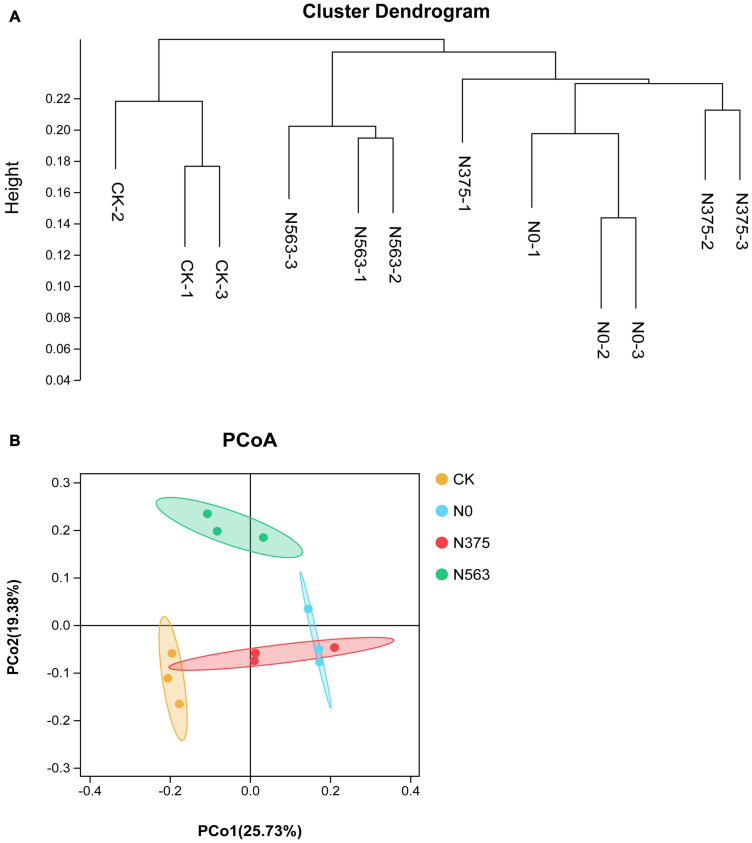
The **(A)** unweighted pair group method with arithmetical averages (UPGMA) cluster analysis and **(B)** principal coordinate analysis of bacterial communities based on OTUs.

An UPGMA cluster dendrogram of bacterial communities was constructed based on the Bray–Curtis distance indices calculated using OTUs to examine the similarity among different treatments (Figure 2A). Grouped together indicated that the HiSeq sequencing technique applied here was robust. Two major clusters could be classified for these 12 soil samples. Cluster 1 consisted of three samples of the CK treatment and was significantly different from the other treatments. Cluster 2 could be grouped into two subclusters. N0 and N375 treatments were clustered into one subcluster and N563 treatments belonged to another subcluster.

Consistent with the hierarchical cluster tree, a two-dimensional PCoA plot based on OTU composition also showed the variations among the four treatments clearly (Figure 2B). The PCoA1 and PCoA2 explained 45.11% of the total bacterial community. The samples treated with different N fertilization regimes separated well, suggesting that different N applications indeed change the soil bacterial community structures. PERMANOVA showed the significant differences in soil bacterial communities among the four treatments (Adonis: *P* = 0.007 < 0.05).

### Soil Bacterial Community Composition

**FIGURE 3:**
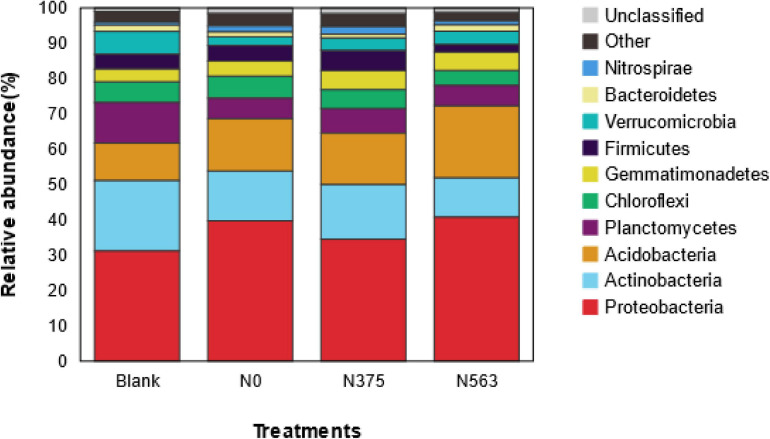
Average relative abundance of the dominant bacteria phyla (relative abundance > 1%) in all treatments. Relative abundances are based on the proportional frequencies of those DNA sequences that could be classified at the phylum.

Based on species annotation and statistical analysis, the sequences were classified into a total of 38 different phyla (1.42% were unclassified at phylum). There were 10 phyla whose relative abundances were more than 1%, as shown in Figure 3. All samples were dominated by the phylum *Proteobacteria*, which accounted for 31.23–40.68% of the total sequences, followed by *Actinobacteria* (11.03–19.93%), *Acidobacteria* (10.67–20.40%), and *Planctomycetes* (5.82–11.45%); *Chloroflexi*, *Gemmatimonadetes*, *Firmicutes*, *Verrucomicrobia*, *Bacteroidetes*, and *Nitrospirae* were abundant (1–10%); and *Chlamydiae*, *Latescibacteria*. *Parcubacteria*, *Saccharibacteria*, *Armatimonadetes*, *Cyanobacteria*, *Elusimicrobia*, *TM6*, and *GAL15* were rare (<1%) (Supplementary Table 1). Obviously, there were some changes in the distribution of phylum as a result of different N application treatments. Compared with CK, N fertilizer application was combined to increase the relative abundance of *Proteobacteria* and *Gemmatimonadetes* and to decrease *Actinobacteria* and *Planctomycetes* (*P* < 0.05), and no statistical changes were found for *Chloroflexi*, *Verrucomicrobia*, and other rare phyla. There was no significant difference on the phyla except for *Elusimicrobia* between the N0 and N375 treatment. Compared with the N375 treatment, the N563 treatment significantly increased the relative abundances of *Proteobacteria*, *Acidobacteria*, and *Bacteroidetes* (*P* < 0.05) and decreased *Firmicutes* (*Bacilli*), *Latescibacteria*, and *Planctomycetes* (*P* < 0.05) (Supplementary Table 1).

The distribution of each class among the different treatments is shown in Table 3 and evaluated by Duncan’s multiple comparison test. The relative abundances of some classes showed no differences, such as Acidi*microbiia*, *Blastocatellia*, *Ktedonobacteria*, and *Spartobacteria*. We can conclude that N375 and N563 treatments significantly decreased the relative abundance of *Betaproteobacteria* but increased *Thermoleophilia* compared with the N0 treatment. Moreover, compared with the N375 treatment, the N563 treatment mainly significantly increased the relative abundance of *Alphaproteobacteria*, *Gammaproteobacteria*, *Acidobacteria*, and *Sphingobacteriia* and decreased *Bacilli.*

**TABLE 3:** Relative abundances (%) of bacterial classes in all treatments.

**Phylum**	**Class**	**CK**	**N0**	**N375**	**N563**
*Proteobacteria*	*Betaproteobacteria*	10.132.29b	22.533.57a	14.554.69b	14.452.34b
	*Alphaproteobacteria*	14.30.52a	9.120.32b	10.421.08b	14.811.43a
	*Deltaproteobacteria*	4.230.70b	5.640.29ab	6.701.71a	5.980.87ab
	*Gammaproteobacteria*	2.530.56b	2.410.06b	2.831.01b	5.501.34a
*Actinobacteria*	*Thermoleophilia*	2.520.32a	1.800.05b	2.220.22a	2.380.04a
	*Actinobacteria*	15.983.86a	10.612.62ab	11.242.38ab	7.261.95b
	*Acidimicrobiia*	0.960.21a	1.230.20a	1.310.54a	1.110.20a
*Acidobacteria*	*Acidobacteria*	4.040.32c	6.872.42b	5.730.94bc	11.250.14a
	*Solibacteres*	1.810.18b	2.280.61ab	2.250.44ab	2.980.10a
	*Blastocatellia*	1.300.47a	1.250.08a	1.560.59a	1.420.08a
	*Holophagae*	0.550.1b	1.050.18a	1.260.34a	1.240.18a
*Planctomycetes*	*Phycisphaerae*	3.780.76a	1.990.07b	2.250.68b	1.910.19b
	*Planctomycetacia*	6.942.30a	3.291.00b	4.092.41ab	3.410.18b
*Chloroflexi*	*Ktedonobacteria*	3.972.08a	3.651.07a	2.611.09a	2.310.37a
*Gemmatimonadetes*	*Gemmatimonadetes*	3.540.48b	4.230.54ab	5.280.78a	5.180.86a
*Firmicutes*	*Bacilli*	3.740.76a	4.000.22a	5.311.5a	1.730.17b
*Verrucomicrobia*	*Spartobacteria*	2.101.00a	0.750.44a	1.641.87a	1.430.53a
*Bacteroidetes*	*Sphingobacteriia*	1.330.23ab	1.070.45b	0.950.02b	1.670.20a
*Nitrospirae*	*Nitrospira*	0.850.22b	1.710.87ab	2.130.59a	1.160.20ab

*Values represent mean ± standard deviation of triplicate measurements.*

*Values within the same row followed by different lowercase letters indicated significant differences (*P* < 0.05) according to Duncan’s multiple comparison tests.*

*CK, without fertilization; N0, without N fertilization; N375, application of 375 kg/ha N; N563, application of 563 kg/ha N.*

At the genus level, the reads represented 483 identifiable genera (59.6% of reads). The relative abundances of the 40 most relatively abundant bacterial genera in all treatments are listed in Supplementary Table 2. The results showed that *Sphingomonas* and *Gemmatimonas* were increased in the N563 treatment, while *Bacillus*, *Nitrospira*, and *Rhizobium*, which are beneficial bacteria, were more abundant in the N375 treatment. We found that N application shifts the N cycle in nitrification, mainly on the *Nitrospira* (Supplementary Table 3), but shows no significant effect on the genera related to nitrogen fixation, methane oxidation, sulfate reduction, and sulfur oxidation (Table 4).

**TABLE 4:** Relative abundance (%) of the functional genera groups in all treatments.

**Function**	**Genera**	**CK**	**N0**	**N375**	**N563**
Nitrogen fixation	*Bradyrhizobium*, *Mesorhizobium*, *Rhizobium*, *Rhizocola*, *Rhizorhapis*	1.190.10a	0.990.14a	1.160.08a	1.060.10a
Nitrification	*Nitrosospira*, *Nitrospira*	0.410.05b	0.440.18b	0.780.18a	0.490.04b
Methane oxidation	*Methylobacterium*, *Methylocaldum*	0.140.01a	0.040.02b	0.070.06b	0.010.00b
Sulfate reduction	*Desulfitibacter*, *Desulfitobacterium*, *Desulfobulbus*, *Desulfosporosinus*, *Desulfovibrio*, *Desulfovirga*	0.030.00a	0.030.00a	0.030.00a	0.030.01a
Sulfur oxidation	*Sulfurifustis*, *Thiobacillus*	0.010.00a	0.030.03a	0.040.04a	0.010.01a

*Values represent mean ± standard deviation of triplicate measurements.*

*Values within the same row followed by different lowercase letters indicated significant differences (*P* < 0.05) according to Duncan’s multiple comparison tests.*

*CK, without fertilization; N0, without N fertilization; N375, application of 375 kg/ha N; N563, application of 563 kg/ha N.*

**FIGURE 4:**
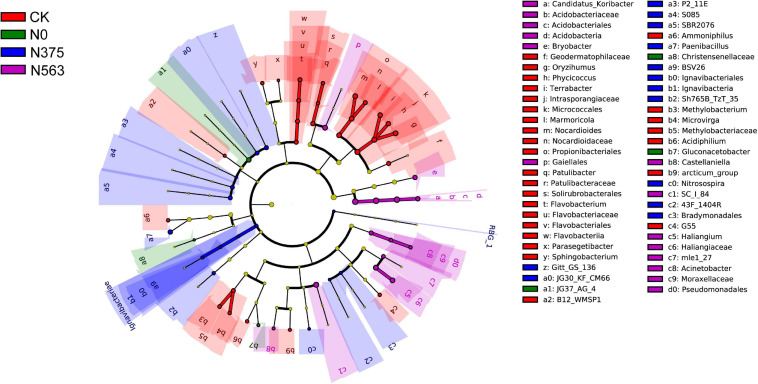
The taxa of bacteria with significantly different abundance in the treatments were identified with the LEfSe method. Colored circles indicate the differentially abundant taxa and the color is corresponding to the different treatments (red for CK, green for N0, blue for N375, and purple for N563). Six rings of the cladogram stand for domain (innermost), phylum, class, order, family, and genus, and lineages with LDA values higher than 2.0 are displayed.

LEfSe analysis showed that significant associations were found among predominant bacterial taxa in the four treatments (Figure 4). LEfSe analysis was conducted to explore which taxa (phylum to genus) were affected by different N applications (Figure 4). Those with an LDA score >2.0 were selected to identify bacterial taxa with statistically significant differences in abundance among treatments. The predominant bacterial taxa were the genera (the relative abundance > 0.1%) *Terrabacter*, *Oryzihumus*, and *Nocardioides* in the CK treatment; *Gluconacetobacter* in the N0 treatment; *Paenibacillus* in the N375 treatment; and *Candidatus_Koribacter*, *Haliangium*, and *Bryobacter* in the N563 treatment.

## Discussion

Soil microorganisms are vital in the agroecosystem environment on account of their important role in cycling mineral compounds, decomposing organic materials, and soil biochemical processes (Li J. G. et al., 2016). In turn, soil microbial biodiversity and functions are affected by various factors, such as soil nutrients, pH, and vegetation. In China, a large area of sugarcane fields was applied with high N fertilization to get higher yields. In our work, a field experiment was conducted to evaluate the impacts of different amounts of N fertilizers on the diversity and community structure in sugarcane soil. Our results showed that higher N fertilization (N563) conversely decreased sugarcane yield than in the N375 treatment, indicating that optimum N application might result in a healthier ecosystem and contribute toward sustainable crop production.

Soil microbial diversity is considered to be critical to integrity, function, and long-term sustainability of soil ecosystems (Kennedy and Smith, 1995). Many studies have shown that greater biodiversity can improve the ecosystem and the stability of microbial functions (Chaer et al., 2009). Therefore, it is critical to maintain and restore the microbial biodiversity in sustainable agriculture systems. Generally, excessive N fertilization decreased the diversity of microbes in bulk and rhizosphere soil (Sun et al., 2019; Wang et al., 2019). In our work, lower ACE, Chao1, and observed species at high N fertilization (N563) compared with those in the N375 and N0 treatments (Table 2) indicated that excessive N fertilization decreased the bacterial species richness, which might result from the selection pressure of high concentrated AN and alteration of soil pH on the microbes (Li J. G. et al., 2016). Moreover, some plant physiological characteristics, such as leaf area index and chlorophyll content, could be regulated by the N level, which could change root exudates or signal of the plant and in turn affect the rhizosphere microbes (Pfenning et al., 2009; Basal and Szabó, 2018).

Soil bacterial diversity and community structures were altered in response to different N application amendments, which agreed with previous studies (Zhang et al., 2017). The relative abundance variation at the phylum level and the LEfSe at each taxonomic level (from phylum to genus) (Figures 3, 4) were conducted to detect microbial population distribution variations among all the treatments. Firstly, hierarchical cluster analysis of similarity and PCoA analysis of the bacterial communities (Figure 2) demonstrated that each treatment formed a unique community structure. *Proteobacteria*, *Acidobacteria*, and *Actinobacteria* were the dominant and responding phyla in all treatments, similar to a previous report in sugarcane soils (Luo et al., 2020). Different *Proteobacteria* have various functions in soil. Generally, *Alphaproteobacteria* and *Gammaproteobacteria* were reported to increase with pH above 4.5 (Rousk et al., 2010), but in our work, N563 treatment with lower pH resulted in a higher abundance of these two subgroups which may be due to a low soil pH below 4.5 (Table 3). Therefore, we speculate that there may be some other factors, such as available N reciprocally affecting these organisms in soil. *Bacteroidetes* was associated with decomposition of recalcitrant carbon compounds, showing positive responses to N fertilization (Yuan et al., 2016). Our results indicated that the relative abundances of *Proteobacteria* and *Bacteroidetes* in N563 treatment were significantly higher than those in N375 treatment (Supplementary Table 1). Similarly, Fierer et al. (2012) discovered that an improved soil N availability increased the abundance of copiotrophic bacterial taxa including *Proteobacteria* and *Bacteroidetes*, which was consistent with our study. *Actinobacteria* was the second abundant phylum and the relative abundance of the N563 treatment was significantly lower than that of the N375 treatment. Chaudhry et al. (2012) reported that high N input can decrease the relative abundance of *Actinobacteria*. Many studies showed that members of *Actinobacteria* could produce the most known antibiotics, such as *tetracycline*, *oxytetracycline*, *gentamicin*, and *streptomycin* and can survive in the soil environment, which are considered to be beneficial in agricultural soils (Polti et al., 2014). Thus, excessive N fertilizer application is not conducive to *Actinobacteria* so as to be harmful to microorganisms in agricultural systems. *Nitrospirae* was most abundant in the N375 treatment, which has been demonstrated to have a positive effect on enhancing the absorption of trace elements from soil to plants and promoting plant growth. That might be one of the reasons for the higher crop yield in the N375 treatment than in the N563 treatment. A recent study based on meta-analysis indicated that both soil microbial diversity and the relative abundances of *Actinobacteria* and *Nitrospirae* were reduced by N fertilizer application, which confirmed our work (Wang et al., 2018).

The application of a large amount of N fertilizers significantly decreased soil pH. This is well-documented and mainly resulted from soil processes which can produce protons, such as nitrification (Geisseler and Scow, 2014). A survey across China indicated that N fertilization application ranging from 8 to 25 years resulted in a decline of soil pH by 0.45–2.20 (Guo et al., 2010). In this work, pH values of the N563 treatment were significantly lower than those of the N375 treatment resulting from overuse of N fertilizer (Larssen and Carmichael, 2000; Guo et al., 2010). Additionally, N fertilization affects soil microbial community *via* changing the chemical properties of soil indirectly (Fierer et al., 2012). We discovered that N fertilizer application significantly impacted soil pH and available N, which indirectly affected microbial communities. *Acidobacteria*, a ubiquitous and abundant member of the soil bacterial community, have been suggested to be closely associated with pH (Jones et al., 2009). In the present study, the highest *Acidobacteria* abundance was in the N563 treatment, probably due to the relatively acidic soil (pH 4.03). Moreover, the responding microbial selected by LEfSe analysis further demonstrated that excessive N fertilization altered the bacterial community, which the genus *Candidatus_Koribacter*, belonging to phylum *Acidobacteria*, was a responding bacterium in the N563 treatment (Figure 4 and Supplementary Table 2).

Changes in bacterial communities reflect the corresponding alterations in functional consequences (Dietrich et al., 2017). Fierer et al. (2012) found that catabolic capabilities of bacterial communities shift across the N gradients, which were significantly correlated with the phylogenetic and metagenomic responses, indicating possible linkages between the structure and functioning of soil microbial communities. In our work, N375 treatment significantly increased the relative abundance of *Nitrospira* related to nitrification but showed no significant effect on the other biogeochemical cycles (Table 4). Moreover, the beneficial bacteria *Bacillus* and *Paenibacillus* were also found most abundant in the N375 treatment. *Bacillus* isolated from rhizospheric soil of sugarcane has N-fixation and biocontrol property against two sugarcane pathogens (Singh et al., 2020). Our results suggested that optimum N application not only reduces cost and waste but also is good for microbials. Overall, our work indicated that N fertilization may change the predominant microbial life-history strategies, preferring a more active microbial community.

## Conclusion

In this work, we examined the effects of different N application rates on microbial diversity and community structure by MiSeq high-throughput sequencing of the 16S rRNA gene. We found that the overuse of N fertilizers significantly decreased pH and increased the available N in soils and obtained a lower yield. N fertilizer application indeed changed the bacterial diversity and community structures in sugarcane soils. Excessive N application significantly decreased the bacterial diversity. The optimum amount of N application could be conducive to beneficial microorganisms, such as *Actinobacteria*, *Nitrospira*, and *Bacillus* and resulted in a healthier ecosystem and higher sustainable crop production.

## Data Availability Statement

The datasets presented in this study can be found in online repositories. The names of the repository/repositories and accession number(s) can be found below: https://www.ncbi.nlm.nih.gov/, SRP269446.

## Author Contributions

YG analyzed the data and wrote the manuscript. JW conceived the study. GL, WC, and YM performed the experiment. SY provided the financial support and technical guidance. All authors contributed to the article and approved the submitted version.

## Conflict of Interest

The authors declare that the research was conducted in the absence of any commercial or financial relationships that could be construed as a potential conflict of interest.

## Publisher’s Note

All claims expressed in this article are solely those of the authors and do not necessarily represent those of their affiliated organizations, or those of the publisher, the editors and the reviewers. Any product that may be evaluated in this article, or claim that may be made by its manufacturer, is not guaranteed or endorsed by the publisher.

## References

[B1] Bao S. D. (2000). *Agrochemical Analysis of Soil.* Beijing: China Agriculture Press.

[B2] Basal O. Szabó A. (2018). The effects of drought and nitrogen on soybean (Glycine max (L.) Merrill) physiology and yield. *Int. J. Agric. Biosyst. Engin.* 12 260–265. 10.5281/zenodo.1474431

[B3] Belén M. A. Mary-Rus M. C. Almudena B. Francisco L. Ana Q. (2016). Liquid organic fertilizers for sustainable agriculture: nutrient uptake of organic versus mineral fertilizers in citrus trees. *PLoS One* 11:e0161619. 10.1371/journal.pone.0161619 27764099 PMC5072554

[B4] Caporaso J. G. Kuczynski J. Stombaugh J. Bittinger K. Bushman F. D. Costello E. K. (2010). QIIME allows analysis of high-throughput community sequencing data. *Nat. Methods* 7 335–336. 10.1038/nmeth.f.303 20383131 PMC3156573

[B5] Chaer G. Fernandes M. Myrold D. Bottomley P. (2009). Comparative resistance and resilience of soil microbial communities and enzyme activities in adjacent native forest and agricultural soils. *Microb. Ecol.* 58 414–424. 10.1007/s00248-009-9508-x 19330551

[B6] Chaudhry V. Rehman A. Mishra A. Chauhan P. S. Nautiyal C. S. (2012). Changes in bacterial community structure of agricultural land due to long-term organic and chemical amendments. *Microb. Ecol.* 64 450–460. 10.1007/s00248-012-0025-y 22419103

[B7] Chen G. Huang J. Fang Y. Zhao Y. Tian X. Jin Y. (2019). Microbial community succession and pollutants removal of a novel carriers enhanced duckweed treatment system for rural wastewater in Dianchi Lake basin. *Bioresour. Technol.* 276 8–17. 10.1016/j.biortech.2018.12.102 30602128

[B8] de Souza R. S. Okura V. K. Armanhi J. Jorrín B. Lozano N. Silva M. (2016). Unlocking the bacterial and fungal communities assemblages of sugarcane microbiome. *Sci. Rep.* 6:28774. 10.1038/srep28774 27358031 PMC4928081

[B9] Dietrich P. Buchmann T. Cesarz S. Eisenhauer N. Roscher C. (2017). Fertilization, soil and plant community characteristics determine soil microbial activity in managed temperate grasslands. *Plant Soil* 419 189–199. 10.1007/s11104-017-3328-4

[B10] dos Santos S. G. da Silva Ribeiro F. Alves G. C. Santos L. A. Reis V. M. (2019). Inoculation with five diazotrophs alters nitrogen metabolism during the initial growth of sugarcane varieties with contrasting responses to added nitrogen. *Plant Soil* 44 363–370. 10.1007/s11104-019-04101-1

[B11] Du Y. Wang T. Wang C. Anane P. S. Liu S. Paz-Ferreiro J. (2019). Nitrogen fertilizer is a key factor affecting the soil chemical and microbial communities in a Mollisol. *Can. J. Microbiol.* 65 510–521. 10.1139/cjm-2018-0683 30901528

[B12] Edgar R. C. (2013). UPARSE: highly accurate OTU sequences from microbial amplicon reads. *Nat. Methods* 10 996–998. 10.1038/nmeth.2604 23955772

[B13] Fierer N. Lauber C. L. Ramirez K. S. Zaneveld J. Bradford M. A. Knight R. (2012). Comparative metagenomic, phylogenetic and physiological analyses of soil microbial communities across nitrogen gradients. *ISME J.* 6 1007–1017. 10.1038/ismej.2011.159 22134642 PMC3329107

[B14] Geisseler D. Scow K. M. (2014). Long-term effects of mineral fertilizers on soil microorganisms–a review. *Soil Biol. Biochem.* 75 54–63. 10.1016/j.soilbio.2014.03.023

[B15] Gu Y. Mi W. H. Xie Y. N. Ma Q. X. Wu L. H. Hu Z. P. (2019). Nitrapyrin affects the abundance of ammonia oxidizers rather than community structure in a yellow clay paddy soil. *J. Soil Sediments* 19 872–882. 10.1007/s11368-018-2075-3

[B16] Guo J. H. Liu X. J. Zhang Y. Shen J. L. Han W. X. Zhang W. F. (2010). Significant acidification in major Chinese croplands. *Science* 327 1008–1010. 10.1126/science.1182570 20150447

[B17] Guo Q. Yan L. Korpelainen H. Niinemets Ü Li C. (2018). Plant-plant interactions and n fertilization shape soil bacterial and fungal communities. *Soil Biol. Biochem* 128 127–138. 10.1016/j.soilbio.2018.10.018

[B46] Jones R. T. Robeson M. S. Lauber C. L. Hamady M. Knight R. Fierer N. (2009). A comprehensive survey of soil acidobacterial diversity using pyrosequencing and clone library analyses. *ISME J.* 3, 442–453. 10.1038/ismej.2008.127 19129864 PMC2997719

[B18] Kennedy A. C. Smith K. L. (1995). Soil microbial diversity and the sustainability of agricultural soils. *Plant Soil* 170 75–86. 10.1007/BF02183056

[B19] Klironomos J. Zobel M. Tibbett M. Stock W. D. Rillig M. C. Parrent J. L. (2011). Forces that structure plant communities: quantifying the importance of the mycorrhizal symbiosis. *New Phytol.* 189 366–370. 10.1111/j.1469-8137.2010.03550.x 21058952

[B20] Larssen T. Carmichael G. R. (2000). Acid rain and acidification in China: the importance of base cation deposition. *Environ. Pollut.* 110 89–102. 10.1016/S0269-7491(99)00279-1 15092859

[B21] Li J. G. Shen M. C. Hou J. F. Li L. Wu J. X. Dong Y. H. (2016). Effect of different levels of nitrogen on rhizosphere bacterial community structure in intensive monoculture of greenhouse lettuce. *Sci. Rep.* 6:25305. 10.1038/srep25305 27121918 PMC4848521

[B22] Li Y. R. Song X. P. Wu J. M. Li C. N. Liang Q. Liu X. H. (2016). Sugar industry and improved sugarcane farming technologies in china. *Sugar Tech.* 18 603–611. 10.1007/s12355-016-0480-8

[B23] Li Y. R. Yang L. T. (2015). Sugarcane agriculture and sugar industry in China. *Sugar Tech*. 17 1–8. 10.1007/s12355-014-0342-1

[B24] Luo J. Lin Z. L. Li S. Y. Que Y. X. Zhang C. F. Yang Z. Q. (2020). Effects of different soil improvement measures on soil physicochemical properties and microbial community structures in mechanically compacted acidified sugarcane field. *Acta Agron. Sin.(China)* 46 596–613.

[B25] Pereira L. B. Andrade G. S. Meneghin S. P. Vicentini R. Ottoboni L. M. M. (2019). Prospecting plant growth-promoting bacteria isolated from the rhizosphere of sugarcane under drought stress. *Curr. Microbiol.* 76 1345–1354. 10.1007/s00284-019-01749-x 31372732

[B26] Pfenning J. Liebig H. P. Graeff S. Claupein W. (2009). Sensor based fine tuning of nitrogen fertilizer applications using spectral feedback signals from tomato plants (Lycopersicon esculentum Mill.). *Acta Hortic*. 824 177–182. 10.17660/ActaHortic.2009.824.20

[B27] Pisa G. Magnani G. S. Weber H. Souza E. M. Faoro H. Monteiro R. A. (2011). Diversity of 16s Rrna genes from bacteria of sugarcane rhizosphere soil. *Braz. J. Med. Biol. Res.* 44 1215–1221. 10.1590/S0100-879X2011007500148 22042267

[B28] Polti M. A. Aparicio J. D. Benimeli C. S. Amoroso M. J. (2014). Role of actinobacteria in bioremediation. *Microbiol. Biodegrad. Biorem* 2014 269–286. 10.1016/B978-0-12-800021-2.00011-X

[B29] Robertson G. P. Vitousek P. M. (2009). Nitrogen in agriculture: balancing the cost of an essential resource. *Annu. Rev. Environ. Resour.* 34 97–125. 10.1146/annurev.environ.032108.105046

[B30] Robinson N. Brackin R. Vinall K. Soper F. Holst J. Gamage H. (2011). Nitrate paradigm does not hold up for sugarcane. *PLoS One* 6:e19045. 10.1371/journal.pone.0019045 21552564 PMC3084252

[B31] Rousk J. Baath E. Brookes P. C. Lauber C. L. (2010). Soil bacterial and fungal communities across a pH gradient in an arable soil. *ISMEJ.* 4 1340–1351. 10.1038/ismej.2010.58 20445636

[B32] Savario C. F. Hoy J. W. (2011). Microbial communities in sugarcane field soils with and without a sugarcane cropping history. *Plant Soil* 341 63–73. 10.1007/s11104-010-0622-9

[B33] Segata N. Izard J. Waldron L. Gevers D. Miropolsky L. Garrett W. S. (2011). Metagenomic biomarker discovery and explanation. *Genome Biol.* 12:R60. 10.1186/gb-2011-12-6-r60 21702898 PMC3218848

[B34] Singh R. Singh P. Haibi L. Song Q. Q. Guo D. J. Solanki M. (2020). Diversity of nitrogen-fixing rhizobacteria associated with sugarcane: a comprehensive study of plant-microbe interactions for growth enhancement in Saccharum Spp. *BMC Plant Biol.* 20:220. 10.1186/s12870-020-02400-9 32423383 PMC7236179

[B35] Singh R. K. Singh P. Li H. B. Yang L. T. Li Y. R. (2017). “Soil–plant–microbe interactions: use of nitrogen-fixing bacteria for plant growth and development in sugarcane,” in *Plant-Microbe Interactions in Agro-Ecological Perspectives*, eds Singh D. Singh H. Prabha R. (Singapore: Springer), 35–39. 10.1007/978-981-10-5813-4_3

[B36] Sun R. Zhang P. Riggins C. R. Zabaloy M. C. Rodríguez-Zas S. Villamil M. B. (2019). Long-term N fertilization decreased diversity and altered the composition of soil bacterial and archaeal communities. *Agronomy* 9:574. 10.3390/agronomy9100574

[B37] Val-Moraes S. P. de Macedo H. S. Kishi L. T. Pereira R. M. Navarrete A. A. Mendes L. W. (2016). Liming in the sugarcane burnt system and the green harvest practice affect soil bacterial community in Northeastern Brazil. *Antonie Van Leeuwenhoek* 109 1643–1654. 10.1007/s10482-016-0764-8 27629424

[B38] Waclawovsky A. J. Sato P. M. Lembke C. G. Moore P. H. Souza G. M. (2010). Sugarcane for bioenergy production: an assessment of yield and regulation of sucrose content. *Plant Biotechnol. J.* 8 263–276. 10.1111/j.1467-7652.2009.00491.x 20388126

[B39] Wang C. Liu D. Bai E. (2018). Decreasing soil microbial diversity is associated with decreasing microbial biomass under nitrogen addition. *Soil Biol. Biochem.* 120 126–133. 10.1016/j.soilbio.2018.02.003

[B40] Wang Q. Ma M. Jiang X. Guan D. Wei D. Zhao B. (2019). Impact of 36 years of nitrogen fertilization on microbial community composition and soil carbon cycling-related enzyme activities in rhizospheres and bulk soils in northeast China. *Appl. Soil Ecol.* 136 148–157. 10.1016/j.apsoil.2018.12.019

[B41] Yang W. Li Z. Wang J. Wu P. Zhang Y. (2013). Crop yield, nitrogen acquisition and sugarcane quality as affected by interspecific competition and nitrogen application. *Field Crops Res.* 146 44–50. 10.1016/j.fcr.2013.03.008

[B42] Yeoh Y. K. Paungfoo-Lonhienne C. Dennis P. G. Robinson N. Ragan M. A. Schmidt S. (2016). The core root microbiome of sugarcanes cultivated under varying nitrogen fertilizer application. *Environ. Microbiol*. 18 1338–1351. 10.1111/1462-2920.12925 26032777

[B43] Yuan X. Knelman J. E. Gasarch E. Wang D. Nemergut D. R. Seastedt T. R. (2016). Plant community and soil chemistry responses to long-term nitrogen inputs drive changes to alpine bacterial communities. *Ecology* 97 1543–1554. 10.1890/15-1160.1 27459784

[B44] Zhang M. Wang W. Zhang Y. Teng Y. Xu Z. (2017). Effects of fungicide iprodione and nitrification inhibitor 3, 4-dimethylpyrazole phosphate on soil enzyme and bacterial properties. *Sci. Total Environ.* 599 254–263. 10.1016/j.scitotenv.2017.05.011 28477482

[B45] Zhang W. F. Dou Z. X. He P. Ju X. T. Powlson D. Chadwick D. (2013). New technologies reduce greenhouse gas emissions from nitrogenous fertilizer in China. *Proc. Natl. Acad. Sci. U.S.A.* 110 8375–8380. 10.1073/pnas.1210447110 23671096 PMC3666697

