# Supplementary material for: Different Amounts of Nitrogen Fertilizer Applications Alter the Bacterial Diversity and Community Structure in the Rhizosphere Soil of Sugarcane
Source: Front Microbiol. 2021 Sep 10;12:721441. doi: 10.3389/fmicb.2021.721441 (PMC8489880; doi:10.3389/fmicb.2021.721441)
Supplement: Supplementary file 1 [file Data_Sheet_1.docx]

**Supporting Information**

**Different Amounts of Nitrogen Fertilizer Applications Alter the Bacterial Diversity and Community Structure in the** **Rhizosphere Soil of Sugarcane**

**Yan Gu^1,†^, Jihua Wang^1,†^, Weijun Cai^2^, Guoliang Li^3^, Yu Mei^1^, Shaohai Yang^1,*^**

^1^Guangdong Provincial Key Laboratory of Crops Genetics and Improvement, Crop Research Institute, Guangdong Academy of Agricultural Sciences, Guangzhou, China

^2^Zhanjiang Academy of Agricultural Sciences, Zhanjiang, China

^3^Institute of Agricultural Resources and Environment, Guangdong Academy of Agricultural Sciences, Guangzhou, China

*** Correspondence:**

Corresponding author: Yang Shaohai

E-mail: yangshaohai@gdaas.cn

^†^ These authors have contributed equally to this work and share first authorship

**Table S1 Relative abundances (%) of bacterial phyla in all treatments. The relative abundances are based on the proportional frequencies of those DNA sequences that could be classified at the phylum level.**

| Phylum | CK | N0 | N375 | N563 |
| --- | --- | --- | --- | --- |
| *Proteobacteria* | 31.23±1.74 c | 39.75±3.42 ab | 34.53±4.45 bc | 40.68±0.75 a |
| *Actinobacteria* | 19.93±3.84 a | 14.11±2.29 ab | 16.43±1.81 a | 11.03±1.86 b |
| *Acidobacteria* | 10.67±1.17 b | 14.74±4.25 b | 14.49±2.39 b | 20.4±1.09 a |
| *Planctomycetes* | 11.45±3.33 a | 5.82±1.00 b | 7.06±3.13 b | 5.84±0.46 b |
| *Chloroflexi* | 5.81±2.23 a | 6.24±1.30a | 5.41±1.24 a | 4.26±0.45 a |
| *Gemmatimonadetes* | 3.61±0.49 b | 4.29±0.53 ab | 5.34±0.72 a | 5.2±0.82 a |
| *Firmicutes* | 4.22±0.80 a | 4.39±0.16 a | 5.73±1.56 a | 2.13±0.12 b |
| *Verrucomicrobia* | 6.43±2.98 a | 2.5±1.21 a | 3.51±3.48 a | 3.84±1.74 a |
| *Bacteroidetes* | 1.75±0.41 a | 1.35±0.39 ab | 1.14±0.01 b | 1.76±0.18 a |
| *Nitrospirae* | 0.85±0.22 b | 1.7±0.84 ab | 2.1±0.59 a | 1.17±0.21 b |
| *Chlamydiae* | 0.7±0.24 a | 0.56±0.11 a | 0.81±0.39 a | 0.42±0.08 a |
| *Latescibacteria* | 0.54±0.19 ab | 0.72±0.08 a | 0.63±0.17 a | 0.35±0.01 b |
| *Parcubacteria* | 0.55±0.39 a | 0.48±0.11 a | 0.54±0.37 a | 0.25±0.12 a |
| *Saccharibacteria* | 0.25±0.11 a | 0.51±0.37 a | 0.32±0.12 a | 0.35±0.12 a |
| *Armatimonadetes* | 0.29±0.13 a | 0.27±0.01 a | 0.2±0.05 a | 0.42±0.21 a |
| *Cyanobacteria* | 0.21±0.02 a | 0.29±0.06 a | 0.26±0.23 a | 0.18±0.04 a |
| *Elusimicrobia* | 0.16±0.03 ab | 0.22±0.06 a | 0.14±0.05 b | 0.11±0.01 b |
| *TM6* | 0.11±0.03 a | 0.12±0.02 a | 0.21±0.13 a | 0.08±0.02 a |
| *GAL15* | 0.05±0.02 a | 0.1±0.03 a | 0.22±0.26 a | 0.09±0.01 a |
| others | 0.16±0.11 | 0.16±0.06 | 0.25±0.16 | 0.1±0.09 |
| Unclassified | 1.03±0.07 | 1.63±0.38 | 1.69±0.36 | 1.32±0.16 |

Values are mean ± standard deviation (n = 3). Values within the same row followed by different lowercase letters indicated significant differences (*P* < 0.05) according to Duncan’s multiple comparison tests. CK, without fertilizers; N0, without N fertilization; N375, application of 375 kg/ha N; N563, application of 563 kg/ha N.

**Table S2****Relative abundances (%) of the 40 most relatively abundant bacterial genera in all treatments.**

| Genus | CK | N0 | N375 | N563 |
| --- | --- | --- | --- | --- |
| *Sphingomonas* | 6.73±1.02 a | 2.28±0.15 b | 3.28±0.83 b | 7.02±1.40 a |
| *Masala* | 2.21±0.60 b | 7.92±0.74 a | 4.6±2.88 b | 3.75±1.55 b |
| *Bacillus* | 3.06±0.78 b | 3.35±0.19 b | 4.35±1.21 a | 1.34±0.16 b |
| *Burkholderia-Paraburkholderia* | 1.27±0.44 b | 5.18±2.31 a | 1.11±0.14 b | 1.11±0.25 b |
| *H16* | 1.21±0.29 b | 1.86±0.58 ab | 2.37±0.54 a | 1.45±0.06 b |
| *Candidatus_Solibacter* | 1.01±0.18 b | 1.23±0.34 b | 1.29±0.29 ab | 1.75±0.18 a |
| *Gemmatimonas* | 1.17±0.09 b | 0.90±0.25 b | 1.04±0.3 b | 1.80±0.09 a |
| *Haliangium* | 0.92±0.09 c | 1.31±0.04 b | 1.11±0.2 bc | 1.56±0.09 a |
| *Oryzihumus* | 2.14±0.52 a | 0.60±0.10 b | 0.88±0.24 b | 0.38±0.17 b |
| *Gemmata* | 1.26±0.30 a | 0.63±0.29 a | 0.84±0.62 a | 0.62±0.07 a |
| *RB41* | 0.60±0.19 a | 0.72±0.04 a | 0.79±0.32 a | 0.79±0.11 a |
| *Planctomyces* | 0.91±0.51 a | 0.55±0.12 a | 0.64±0.39 a | 0.72±0.12 a |
| *Bradyrhizobium* | 0.70±0.17 a | 0.62±0.04 a | 0.67±0.08 a | 0.82±0.07 a |
| *Nocardioides* | 1.86±0.56 a | 0.20±0.02 b | 0.38±0.14 b | 0.21±0.04 b |
| *Bryobacter* | 0.49±0.05 b | 0.59±0.15 b | 0.67±0.14 b | 0.89±0.06 a |
| *Rhodanobacter* | 0.27±0.06 a | 0.22±0.05 a | 0.41±0.51 a | 1.52±1.25 a |
| *Streptomyces* | 0.65±0.48 a | 0.45±0.18 a | 0.83±0.18 a | 0.34±0.08 a |
| *Anaeromyxobacter* | 0.33±0.11 b | 0.52±0.18 ab | 0.85±0.20 a | 0.47±0.23 b |
| *Candidatus_Koribacter* | 0.35±0.08 b | 0.33±0.09 b | 0.50±0.15 b | 0.86±0.1 a |
| *Nitrospira* | 0.41±0.05 b | 0.44±0.18 b | 0.70±0.26 a | 0.49±0.04 b |
| *11-24* | 0.45±0.30 a | 0.40±0.09 a | 0.63±0.27 a | 0.35±0.02 a |
| *Geobacter* | 0.30±0.09 a | 0.35±0.06 a | 0.72±0.65 a | 0.39±0.31 a |
| *Phenylobacterium* | 0.44±0.16 ab | 0.37±0.04 b | 0.28±0.11 b | 0.66±0.21 a |
| *Terrabacter* | 1.14±0.35 a | 0.16±0.03 b | 0.29±0.16 b | 0.08±0.02 b |
| *Terracidiphilus* | 0.27±0.08 b | 0.26±0.05 b | 0.24±0.10 b | 0.53±0.03 a |
| *Paenibacillus* | 0.2±0.03 b | 0.31±0.04 ab | 0.57±0.31 a | 0.14±0.09 b |
| *Acidothermus* | 0.46±0.41 a | 0.23±0.06 a | 0.22±0.05 a | 0.23±0.05 a |
| *Roseiflexus* | 0.34±0.09 a | 0.24±0.1 ab | 0.39±0.06 a | 0.16±0.04 b |
| *Zavarzinella* | 0.41±0.11 a | 0.16±0.04 b | 0.23±0.17 ab | 0.31±0.05 ab |
| *Cupriavidus* | 0.38±0.06 a | 0.24±0.05 ab | 0.28±0.15 a | 0.1±0.06 b |
| *Pirellula* | 0.40±0.22 a | 0.19±0.07 ab | 0.18±0.11 ab | 0.13±0.06 b |
| *Sorangium* | 0.20±0.02 a | 0.27±0.05 a | 0.18±0.06 a | 0.26±0.04 a |
| *Gaiella* | 0.21±0.06 b | 0.14±0.03 b | 0.34±0.07 a | 0.17±0.07 b |
| *Catenulispora* | 0.12±0.04 b | 0.23±0.07 ab | 0.20±0.01 ab | 0.31±0.13 a |
| *Aquicella* | 0.15±0.07 a | 0.28±0.04 a | 0.25±0.12 a | 0.16±0.03 a |
| *Rhizobium* | 0.28±0.01 a | 0.16±0.05 b | 0.26±0.08 a | 0.10±0.02 b |
| *Tumebacillus* | 0.25±0.14 a | 0.21±0.04 a | 0.23±0.09 a | 0.12±0.04 a |
| *Phycicoccus* | 0.52±0.17 a | 0.09±0.02 b | 0.14±0.08 b | 0.05±0.01 b |
| *Jatrophihabitans* | 0.22±0.03 ab | 0.16±0.02 bc | 0.14±0.03 c | 0.26±0.06 a |
| *Sinomonas* | 0.18±0.08 a | 0.26±0.08 a | 0.26±0.16 a | 0.07±0.02 a |

Values are mean ± standard deviation (n = 3). Values within the same row followed by different lowercase letters indicated significant differences (*P* < 0.05) according to

Duncan’s multiple comparison tests. CK, without fertilizers; N0, without N fertilization; N375, application of 375 kg/ha N; N563, application of 563 kg/ha N.

**Table S3 Relative abundance (%) of functional genera in all the treatments.**

| Function | Genus | CK -1 | CK -2 | CK -3 | N0-1 | N0-2 | N0-3 | N375-1 | N375-2 | N375-3 | N563-1 | N563-2 | N563-3 |
| --- | --- | --- | --- | --- | --- | --- | --- | --- | --- | --- | --- | --- | --- |
| Nitrogen fixation | *Bradyrhizobium* | 0.70 | 0.52 | 0.86 | 0.58 | 0.64 | 0.66 | 0.65 | 0.76 | 0.60 | 0.80 | 0.90 | 0.76 |
|  | *Mesorhizobium* | 0.18 | 0.29 | 0.16 | 0.14 | 0.25 | 0.19 | 0.22 | 0.23 | 0.18 | 0.16 | 0.13 | 0.09 |
|  | *Rhizobium* | 0.30 | 0.29 | 0.27 | 0.11 | 0.18 | 0.20 | 0.21 | 0.22 | 0.36 | 0.08 | 0.12 | 0.11 |
|  | *Rhizocola* | 0.00 | 0.00 | 0.00 | 0.00 | 0.01 | 0.00 | 0.00 | 0.02 | 0.00 | 0.00 | 0.00 | 0.00 |
|  | *Rhizorhapis* | 0.00 | 0.00 | 0.00 | 0.00 | 0.00 | 0.00 | 0.00 | 0.01 | 0.01 | 0.00 | 0.01 | 0.02 |
| Nitrification | *Nitrosospira* | 0.00 | 0.00 | 0.00 | 0.00 | 0.00 | 0.00 | 0.02 | 0.01 | 0.01 | 0.01 | 0.00 | 0.01 |
|  | *Nitrospira* | 0.44 | 0.42 | 0.36 | 0.64 | 0.35 | 0.32 | 0.97 | 0.64 | 0.70 | 0.44 | 0.51 | 0.52 |
| Methane oxidation | *Methylobacterium* | 0.15 | 0.13 | 0.14 | 0.02 | 0.05 | 0.04 | 0.01 | 0.06 | 0.13 | 0.01 | 0.01 | 0.00 |
|  | *Methylocaldum* | 0.00 | 0.00 | 0.00 | 0.00 | 0.00 | 0.00 | 0.00 | 0.00 | 0.00 | 0.00 | 0.00 | 0.00 |
| Sulfate reduction | *Desulfitibacter* | 0.00 | 0.00 | 0.00 | 0.00 | 0.00 | 0.00 | 0.01 | 0.00 | 0.00 | 0.00 | 0.00 | 0.00 |
|  | *Desulfitobacterium* | 0.02 | 0.01 | 0.00 | 0.00 | 0.00 | 0.00 | 0.00 | 0.00 | 0.00 | 0.00 | 0.00 | 0.00 |
|  | *Desulfobacca* | 0.00 | 0.00 | 0.00 | 0.00 | 0.00 | 0.00 | 0.00 | 0.00 | 0.00 | 0.00 | 0.00 | 0.00 |
|  | *Desulfobulbus* | 0.00 | 0.01 | 0.00 | 0.00 | 0.00 | 0.00 | 0.01 | 0.01 | 0.00 | 0.00 | 0.01 | 0.00 |
|  | *Desulfosporosinus* | 0.01 | 0.00 | 0.00 | 0.01 | 0.01 | 0.00 | 0.01 | 0.00 | 0.00 | 0.00 | 0.01 | 0.00 |
|  | *Desulfovibrio* | 0.02 | 0.01 | 0.00 | 0.00 | 0.03 | 0.01 | 0.02 | 0.02 | 0.00 | 0.01 | 0.02 | 0.00 |
|  | *Desulfovirga* | 0.01 | 0.03 | 0.00 | 0.01 | 0.00 | 0.00 | 0.01 | 0.01 | 0.01 | 0.01 | 0.02 | 0.00 |
|  | *Dethiobacter* | 0.00 | 0.00 | 0.00 | 0.00 | 0.00 | 0.00 | 0.00 | 0.00 | 0.00 | 0.00 | 0.00 | 0.00 |
| Sulfur oxidation | *Sulfurifustis* | 0.00 | 0.01 | 0.01 | 0.00 | 0.06 | 0.02 | 0.07 | 0.04 | 0.00 | 0.01 | 0.00 | 0.00 |
|  | *Thiobacillus* | 0.00 | 0.00 | 0.00 | 0.00 | 0.00 | 0.00 | 0.00 | 0.01 | 0.00 | 0.00 | 0.01 | 0.00 |

Values are mean ± standard deviation (n = 3). Values within the same row followed by different lowercase letters indicated significant differences (*P* < 0.05) according to Duncan’s multiple comparison tests. CK, without fertilizers; N0, without N fertilization; N375, application of 375 kg /ha N; N563, application of 563 kg /ha N.
